# Cardiovascular Risk During the 90-Day Vulnerable Window After COPD Exacerbations: A Narrative Review

**DOI:** 10.3390/life16060999

**Published:** 2026-06-14

**Authors:** Dana-Maria Avasilcăi, Florin-Dumitru Mihălţan

**Affiliations:** 1 Department of Cardio-Thoracic Pathology, “Carol Davila” University of Medicine and Pharmacy, 050474 Bucharest, Romania; florin.mihaltan@umfcd.ro; 2 Institute of Pneumology “Marius Nasta”, 050159 Bucharest, Romania

**Keywords:** chronic obstructive pulmonary disease, COPD exacerbations, cardiovascular risk, MACE, post-exacerbation period, systemic inflammation, cardiopulmonary interactions

## Abstract

Chronic obstructive pulmonary disease (COPD) extends beyond the respiratory system and is closely linked to an increased risk of cardiovascular complications. Exacerbations represent critical periods of cardiovascular vulnerability, with a marked rise in major adverse cardiovascular events observed in the early post-exacerbation phase. This narrative review synthesizes current evidence on the epidemiology, pathophysiological mechanisms, and therapeutic implications of cardiovascular risk following COPD exacerbations. A structured literature search was conducted to identify relevant studies in this setting. Cardiovascular risk is elevated following exacerbations, particularly within the first weeks, and remains increased for months thereafter. Multiple pathophysiological mechanisms contribute to this vulnerable window. Systemic inflammation, marked by elevated cytokines such as IL-6, IL-8, and CRP, promotes endothelial dysfunction, vascular oxidative stress, and impaired nitric oxide bioavailability. Despite the well-established link, cardiovascular disease remains overlooked and undertreated in patients with COPD, and the use of guideline-directed cardiovascular therapies is suboptimal. A more systematic, integrated approach to cardiovascular assessment and management in patients with COPD is warranted to improve outcomes.

## 1. Introduction

Chronic obstructive pulmonary disease (COPD) is now understood as a systemic inflammatory disorder with significant extrapulmonary manifestations. Among these, cardiovascular disease is one of the most prevalent and clinically relevant comorbidities, with major adverse cardiovascular events (MACE), including myocardial infarction, stroke, and cardiovascular death, accounting for a substantial proportion of mortality in this population [1]. The association between COPD and cardiovascular disease persists even after adjustment for traditional risk factors, suggesting a disease-specific mechanism and a complex interplay between the two conditions [2].

According to the Global Initiative for Chronic Obstructive Lung Disease (GOLD), an exacerbation of COPD (ECOPD) is defined as an acute deterioration of respiratory symptoms over a short period, typically ≤14 days, and is classified as mild, moderate, or severe, each category carrying distinct prognostic implications [3]. Beyond their respiratory impact, exacerbations are increasingly recognized as critical periods of heightened cardiovascular risk [4]. This risk is strongly influenced by exacerbation severity, with severe events associated with a substantially greater increase in cardiovascular complications compared to moderate episodes. For instance, the risk of acute myocardial infarction has been recorded to increase eight-fold following severe exacerbations, compared to a more modest rise after moderate events [5].

Large longitudinal studies further demonstrated a pronounced temporal relationship between exacerbations and cardiovascular events. Cardiovascular risk peaks immediately after an exacerbation, particularly within the first two weeks, and, although it gradually declines, remains significantly elevated beyond one year [6]. The magnitude and timing of this risk differ according to exacerbation severity, with severe events associated with an early and marked increase, whereas moderate exacerbations show a more delayed peak. Consistently, both moderate and severe exacerbations are linked to a sustained increase in cardiovascular morbidity and mortality, underscoring exacerbations as pivotal triggers of adverse cardiovascular outcomes [7].

Cardiovascular events occurring during exacerbations are frequently underdiagnosed in clinical practice, as overlapping symptoms such as dyspnea and chest discomfort may obscure their identification [8]. This diagnostic uncertainty can delay appropriate management and may contribute to worse outcomes. Moreover, patients with COPD are less likely to receive guideline-directed cardiovascular therapies compared with individuals without COPD which may partly explain the persistence of elevated cardiovascular risk and accelerated disease progression [9].

The relationship between COPD and cardiovascular disease is only partially explained by shared risk factors such as smoking, physical inactivity, and environmental exposures. Smoking, the principal risk factor for COPD, remains a major cause of preventable mortality worldwide and is associated with a substantial reduction in life expectancy compared with non-smokers [10]. Emerging evidence suggests that both conditions are linked through overlapping pathophysiological mechanisms. COPD has been conceptualised as part of a broader systemic process involving chronic inflammation, endothelial dysfunction, and accelerated ageing, all of which contribute to the development and progression of atherosclerotic disease. In this context, COPD may act not only as a comorbidity but also as an independent contributor to cardiovascular risk [11].

This narrative review aims to provide a comprehensive overview of the epidemiology, pathophysiological mechanisms, and therapeutic implications of cardiovascular risk following COPD exacerbation, with a particular focus on missed opportunities for cardiovascular risk assessment and management.

## 2. Materials and Methods

We conducted this narrative review to evaluate the impact of COPD exacerbations on cardiovascular risk. Literature search was performed using electronic databases, such as PubMed, ScienceDirect and Google Scholar between January and March 2026. Search terms included combinations of keywords related to “chronic obstructive pulmonary disease”, “COPD exacerbations”, “cardiovascular disease”, “major adverse cardiovascular events”, “myocardial infarction”, “stroke”, “heart failure”, “inflammation”, and “COPD treatment”. Eligible studies included original research articles, randomized controlled trials, observational studies, and relevant systematic reviews published in English. Priority was given to recent literature published between 2018 and 2026, while earlier studies were selectively included where necessary to support established pathophysiological concepts or theoretical background.

Inclusion criteria comprised studies involving the adult population with a confirmed diagnosis of COPD, addressing the association between COPD exacerbation and cardiovascular outcomes, including ischemic heart disease, heart failure, atrial fibrillation or other arrhythmias, as well as studies evaluating therapeutic strategies targeting COPD and cardiovascular comorbidities.

Exclusion criteria included non-English publications, lack of full-text availability, case reports or case series, letters to the editor, studies involving pediatric population, unclear COPD diagnosis, or mixed population (e.g., COPD-asthma overlap) without separate analysis. Studies focusing exclusively on pulmonary function or exacerbation frequency, without reporting cardiovascular outcomes, were also excluded.

**Figure 1:**
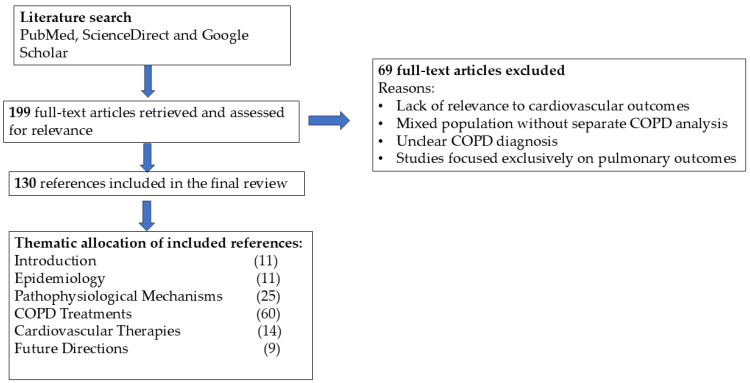
Study selection process and thematic allocation of included references. A total of 199 full-text articles were assessed for relevance, of which 130 references were included in the final review. A total of 69 full-text articles were excluded based on predefined exclusion criteria. Included references were allocated according to their primary thematic contribution. References contributing to multiple sections were assigned once to the subsection in which they provided the main supporting evidence.

Additional relevant studies were identified through manual screening of the reference lists of selected articles. Articles were initially screened for relevance based on title and abstract, and potentially relevant publications were retrieved in full text for detailed evaluation. A total of 199 full-text articles were assessed for relevance, of which 130 articles were ultimately included in the final review (Figure 1). Study selection and synthesis were guided by relevance to the predefined themes of the review, including epidemiology, pathophysiological mechanisms, COPD treatments, cardiovascular therapies, and future directions. Priority was given to studies providing evidence on the association between COPD exacerbations and cardiovascular outcomes, as well as therapeutic strategies with potential implications for cardiovascular risk. Given the narrative nature of this review, no formal quality assessment or meta-analysis was performed.

## 3. Epidemiology of Cardiovascular Events After ECOPD

Within the epidemiology of COPD, cardiovascular disease remains the leading cause of non-respiratory death [3,6,7,12]. A pivotal concept in assessing this risk is major adverse cardiovascular events. Functioning as a composite endpoint, MACEs typically encompass non-fatal myocardial infarction, non-fatal stroke and cardiovascular death [13]. Epidemiological data indicate that MACE risk is not uniformly distributed but rather exhibits a critical maximum immediately following the onset of an exacerbation. Using a self-controlled case series, Rothnie et al. showed that the risk of acute myocardial infarction (AMI) is nearly doubled (IRR 1.96; 95% CI 1.52–2.52) within 1–3 days following a moderate exacerbation and increases eightfold after a severe event, remaining 65% higher during the first 91 days compared with stable disease periods [14]. These findings support a dose–response relationship between exacerbation frequency and cardiovascular risk: while a single moderate exacerbation was not associated with a significantly increased risk of myocardial infarction in a large register-based cohort study in Sweden [15]; the risk increased substantially with recurrent events, rising by 57% in patients with ≥2 moderate exacerbations and by 47% after one severe exacerbation, reaching 82% in those with multiple severe events, further supporting the cumulative cardiovascular impact of exacerbation burden. The impact of exacerbation-related myocardial vulnerability extends beyond AMI incidence to clinical outcomes. In a population-based cohort of over 26,000 COPD patients with first-time AMI, prior hospitalization for ECOPD within the preceding year was independently associated with a 33% higher 90-day mortality risk and a 23% increase in overall mortality. The highest mortality burden was observed in patients with two or more prior exacerbation-related hospitalizations, indicating that exacerbation frequency not only precipitates myocardial events but also worsens their prognosis [16].

This temporal pattern extends beyond myocardial infarction to other cardiovascular events. Stroke, another major component of MACEs, also shows a marked early peak, highlighting a critical window of cerebrovascular vulnerability following exacerbation. Rothnie et al. extended their analysis to ischemic stroke, identifying 3466 COPD patients who experienced at least one exacerbation and subsequently a first ischemic stroke during follow-up. Stroke risk exhibited a similar but temporally distinct pattern compared with myocardial infarction, peaking in the 4–7 days after exacerbation onset (Table 1), with an 83% increase following moderate exacerbations and a nearly fourfold increase after severe events. This excess risk persisted beyond the acute phase, remaining elevated throughout the first 91 days. In contrast to myocardial infarctions, where risk declines toward baseline after around 28 days, stroke risk appears to remain elevated for longer, suggesting potentially different underlying mechanisms [14]. A large self-controlled case series using population-based data from over 362,000 COPD patients across three US states reported similar findings, with ischemic stroke incidence significantly higher in the 30-day period following ECOPD compared with the pre-exacerbation period (IRR 1.77; 95% CI 1.55–2.01). The excess risk peaked within the first 90 days after exacerbation and declined gradually thereafter, supporting a defined period of excess stroke risk following ECOPD [17]. Similarly, as observed for myocardial infarction, prior hospitalization for ECOPD not only raises the incidence of ischemic stroke but also worsens outcomes. In the same Taiwanese population-based cohort, COPD patients with a prior acute exacerbation hospitalization within the preceding year experienced a 46% higher risk of 90-day mortality and a 29% higher overall mortality following ischemic stroke, with the highest mortality observed in patients with two or more prior exacerbation-related hospitalizations [16].

Following the increased risk of myocardial infarction and stroke, cardiovascular death represents the most severe manifestation within the spectrum of major adverse cardiovascular events associated with COPD exacerbations. Although data specifically addressing cardiovascular mortality remain limited compared with non-fatal events, available evidence indicates that exacerbations significantly contribute to short-term cardiovascular mortality risk. A nationwide case-crossover study from Denmark including 118,807 patients who experienced a MACE after an acute COPD exacerbation reported that cardiovascular death accounted for 24.8% of events, second only to myocardial infarction. Moreover, the risk of cardiovascular death increased more than fourfold in the first 4 weeks after exacerbation onset [18]. The EXAcerbations of Chronic obstructive lung disease and their OutcomeS—Cardiovascular (EXACOS-CV) US study, a large retrospective cohort, showed a 30-day hazard ratio for cardiovascular death of 1.63 (95% CI 1.14–2.35) following a first exacerbation compared with non-exacerbating patients. Mortality increased with recurrent exacerbations, with the hazard of all-cause death exceeding threefold after a second exacerbation (HR 3.35; 95% CI 2.52–4.46) and remaining similarly elevated after a third (HR 3.40; 95% CI 2.20–5.24). In addition, severe exacerbations were associated with higher short-term mortality than moderate events (HR 5.09 versus 1.22 at 30 days), further emphasizing the impact of exacerbation severity on fatal outcomes [19].

A broader spectrum of cardiovascular complications has also been described following COPD exacerbations, extending outside the traditional MACE definition. Arrhythmias occur in approximately 15% of patients hospitalized for ECOPD and are associated with a more than threefold increase in in-hospital mortality, with atrial fibrillation (AF) conferring an even higher mortality risk [20]. In addition, heart failure (HF) and atrial fibrillation show a marked increase in the early post-exacerbation period, as shown in a large population-based study, where the risk of incident hospitalization for HF and AF was approximately sixfold higher within the first 30 days after an exacerbation compared with non-exposed patients [21]. Among individuals with pre-existing atrial fibrillation, ECOPD has also been associated with an increased rate of AF-related emergency department visits and hospitalizations, nearly doubling within the first 90 days after exacerbation before returning to baseline levels, suggesting that ECOPD acts as a transient trigger for arrhythmic instability [22]. Pulmonary embolism (PE) further contributes to this spectrum, with exacerbation frequency and severity associated with a progressively increased long-term risk of PE, reaching more than a twofold increase in patients with recurrent severe exacerbations, with the association being most pronounced during the first year of follow-up and gradually decreasing thereafter [15].

**Table 1:** Temporal pattern of cardiovascular risk following COPD exacerbations.

Time Interval After Exacerbation	Cardiovascular Events	Estimated Risk	Key Observations/Clinical Implications	Reference
1–3 days	AMI *	IRR 1.96 (95% CI 1.52–2.52) for moderate exacerbations; IRR 8.00 (95% CI 5.81–11.01) for severe exacerbations	Represents the period of highest acute cardiovascular instability, likely driven by abrupt inflammatory and hemodynamic changes	[14]
4–7 days	Ischemic stroke	IRR 1.83 (95% CI 1.38–2.42) for moderate exacerbations; IRR 3.84 (95% CI 1.99–7.42) for severe exacerbations	Slightly delayed peak compared with AMI, suggesting distinct pathophysiological triggers	[14]
1–30 days	HF	aOR 6.25 (95% CI 5.10–7.66) after severe exacerbation;	High burden of arrhythmias and decompensated heart failure; reflects autonomic imbalance and hemodynamic stress	[21]
	AF	aOR 5.78 (95% CI 4.45–7.50) after severe exacerbation		
	CV death	OR 4.33 (95% CI 4.15–4.52)	Major contributor to early mortality; highlights need for close monitoring during acute and early recovery phase	[18]
Overall (0–90 days)	AMI	IRR 1.65 (95% CI 1.50–1.81) compared to stable periods;	Defines the vulnerable window of sustained cardiovascular risk following exacerbation	[14]
	Ischemic stroke	IRR 1.51 (95% CI 1.39–1.65) compared to stable periods		
	AF-related hospitalization	RR 1.93 (95% CI 1.63–2.29), returning to baseline levels within 180 days	Risk persists beyond acute phase; supports the need for continued surveillance after discharge	[22]
>90 days to 1 year	Recurrent CV events and mortality	HR ~1.1–1.3; Risk remains modestly elevated beyond 90 days, with gradual decline toward baseline. This is supported by interval-specific estimates of HR 1.08 (91 days-6 months) and HR 1.14 (6–12 months)	Persistent but attenuated cardiovascular risk beyond 90 days highlights the importance of long-term monitoring and optimization of cardiovascular risk factors, especially in patients with recurrent exacerbations	[19]

* AMI—Acute Myocardial Infarction; IRR—Incidence Rate Ratio; RR—Relative Risk; CI—Confidence Interval; aOR—Adjusted Odds Ratio; CV—Cardiovascular; HF—Heart Failure; AF—Atrial Fibrillation; HR—Hazard Ratio.

## 4. Pathophysiological Mechanisms During the Vulnerable Window

COPD is associated with an elevated baseline cardiovascular risk profile, likely driven by chronic systemic inflammation, oxidative stress, endothelial dysfunction, and shared risk factors such as smoking, physical inactivity and advancing age [23]. In addition, disease-specific pathophysiological alterations such as increased susceptibility to hypoxemia, elevated resting heart rate and impaired autonomic regulation, further contribute to cardiovascular vulnerability [24]. Together, these changes may contribute to the elevated cardiovascular risk observed in COPD and support its role as a potential independent cardiovascular risk factor [24]. These mechanisms contribute to the development and progression of cardiovascular comorbidities even in stable disease [25]. However, during exacerbations, these underlying processes are markedly amplified, giving rise to a transient “vulnerable window” during which the likelihood of acute cardiovascular events is substantially increased [26].

Systemic inflammation represents a central mechanism linking ECOPD to cardiovascular events. The precise origin of systemic inflammation in COPD remains incompletely understood, with both the translocation of inflammatory mediators into the bloodstream and that of a parallel systemic inflammatory response being proposed [27]. Chronic inflammation in the lungs is characterized by the accumulation of large numbers of alveolar macrophages, neutrophils, and T and B lymphocytes, which release pro-inflammatory cytokines locally in response to inhaled cigarette smoke and other respiratory pollutants, contributing to airway remodeling [28]. Importantly, similar inflammatory processes are also observed in never-smokers with COPD, where exposures such as biomass smoke, air pollution, or early-life lung injury may drive chronic airway inflammation, often manifesting in distinct inflammatory phenotypes (e.g., neutrophilic, eosinophilic, or mixed), which contribute to disease heterogeneity and differential treatment responses [29]. The effects of these inflammatory mediators are not confined to the lungs but extend systemically, where they contribute to the progression of pre-existing cardiovascular comorbidities and accelerate atherosclerotic plaque development [30]. Furthermore, COPD exacerbations are associated with increased plasma levels of inflammatory biomarkers, including C-reactive protein (CRP) and interleukin-6 and interleukin-8 (IL-6 and IL-8) [31], which may further exacerbate endothelial dysfunction, promote plaque instability and enhance a prothrombotic state, thereby acting as triggers for acute myocardial infarction [32]. As a result, this inflammatory surge may contribute to the increased incidence of acute cardiovascular events observed in the early post-exacerbation period.

Oxidative stress in COPD arises from both exogenous sources, such as cigarette smoke, and endogenous production of reactive oxygen species (ROS) by activated inflammatory cells. COPD patients also show reduced antioxidant capacity, partly due to depletion of protective systems such as glutathione enzymes [33]. The resulting excess of ROS promotes oxidative modification of DNA, lipids, and proteins, contributing to cellular injury and perpetuation of inflammatory signaling pathways. At the vascular level, ROS reduce nitric oxide (NO) bioavailability and induce endothelial dysfunction, leading to vascular remodeling, arterial stiffness and the progression of atherosclerosis [34]. Oxidative stress is closely intertwined with hypoxia, as reduced oxygen availability further enhances ROS production and amplifies vascular injury. Under hypoxic conditions, cellular adaptation is mediated by activation of hypoxia-inducible factor-1 (HIF-1), which coordinates responses to reduced oxygen levels, including angiogenesis, erythropoiesis and apoptosis [35]. These processes may persist beyond the acute phase of exacerbations, promoting sustained vascular impairment, plaque destabilization and a prothrombotic milieu, thereby providing a mechanistic basis for the heightened incidence of cardiovascular events observed in the weeks following an exacerbation [36].

Stable COPD is independently associated with impaired endothelial function, as evidenced by reduced vasodilatory responses in both conduit arteries and the microvasculature when compared to non-COPD controls [37]. Exacerbations further intensify the pathophysiological processes present in stable disease. The acute spike in inflammatory mediators, heightened oxidative stress, acute hypoxia and sympathetic nervous system overdrive collectively suppress endothelial nitric oxide synthase activity and reduce vascular smooth muscle responsiveness, creating an environment that promotes endothelial injury and increases cardiovascular risk [38]. In parallel, this injured endothelium acquires prothrombotic properties, reflected by elevated circulating biomarkers such as fibrinogen and von Willebrand factor, favoring coagulation pathway engagement [39]. In this context, endothelial activation, particularly through increased von Willebrand factor release, facilitates platelet adhesion to the vascular wall, while enhanced thrombin generation further amplifies platelet activation [40]. Together, these processes reflect a coordinated interplay among the endothelium, platelets, and coagulation pathways, resulting in a systemic prothrombotic state during ECOPD that may precipitate ischemic events such as myocardial infarction and stroke [41]. In line with these findings, a prospective observational study in the Netherlands further demonstrated that, beyond serving as markers of platelet activation, elevated platelet–monocyte complexes (PMCs) during exacerbations of COPD also have functional implications, as platelet hyperreactivity at admission has been associated with an increased risk of exacerbation relapse, highlighting a bidirectional relationship between coagulation dysregulation and disease recurrence [42].

In addition to vascular and hemostatic alterations, autonomic nervous system imbalance constitutes an important mechanism contributing to cardiovascular risk in COPD. While systemic parasympathetic tone is generally reduced, enhanced cholinergic activity at the airway level contributes to bronchoconstriction, whereas beta-2-adrenergic receptor desensitization may further impair bronchodilatation, promoting airflow limitation and increasing susceptibility to exacerbations [43]. Chronic hypoxia plays a central role in this process by stimulating sympathetic nervous system activity through adrenergic pathways, thereby promoting atrial ectopy and the development of an arrhythmogenic substrate [44]. During exacerbations, airway obstruction intensifies this imbalance by inducing carbon dioxide retention and hypoxemia, which activate carotid body chemoreceptors and pulmonary stretch receptors, leading to increased sympathetic activity and reduced vagal tone [45]. Consistent with these alterations, cardiac autonomic dysregulation is well-recognized in COPD and is characterized by resting tachycardia, attenuated heart rate variability, reduced baroreflex sensitivity, increased sympathetic nerve activity, and abnormal heart rate recovery after exertion, reflecting a shift toward sustained sympathetic dominance and impaired parasympathetic responsiveness compared with healthy controls [46]. In patients with COPD, reduced heart rate variability not only reflects cardiac autonomic dysfunction but may also act as an early indicator of malignant arrhythmias and sudden cardiac death [47].

## 5. Impact of COPD Treatments During and After Exacerbations

Given the marked cardiovascular vulnerability during the early post-exacerbation period, therapies used in this setting must be considered not only in terms of respiratory benefit but also their potential to modulate cardiovascular risk (Table 2). The management of COPD exacerbations during and after hospitalization is traditionally focused on relieving airflow limitation and preventing early relapse. Although severe exacerbations requiring hospitalization attract the greatest clinical attention, most ECOPD episodes are managed in the outpatient setting [3]. This broad clinical burden is significant because the mainstay therapies, specifically high-dose beta-2-agonists and systemic corticosteroids, may escalate cardiovascular risk. Through mechanisms such as sympathetic overactivation, metabolic disturbance, and fluid retention, these treatments can trigger arrhythmogenesis or atrial fibrillation [44]. Consequently, therapies used to stabilize respiratory function may exert complex and sometimes opposing cardiovascular effects, reflecting a therapeutic trade-off during the vulnerable early post-exacerbation period.

### 5.1. Short-Acting Bronchodilators

GOLD guidelines recommend short-acting beta-2-agonists (SABAs) as first-line bronchodilator therapy in moderate to severe COPD exacerbations [3]. Despite their rapid bronchodilatory effects and symptomatic benefit, high-dose administration may lead to significant systemic absorption, reducing lung selectivity. Their sympathomimetic activity can induce acute increases in heart rate, with transient chronotropic effects observed following inhalation [48]. In addition, inhaled salbutamol has been shown to decrease atrial refractoriness, potentially contributing to higher prevalence of atrial fibrillation in patients with COPD [44]. Moreover, a retrospective cohort study conducted in Canada, found a dose–response relationship between SABA exposure and adverse outcomes, with increasing canister use associated with progressively higher risks of major cardiovascular events within 90-day follow up period [49]. Importantly, this relationship does not appear to be entirely driven by exacerbation burden, as frequent SABA use has been associated with a significantly increased incidence of acute cardiovascular events even in patients without prior exacerbations [50]. Similarly, a twofold increased risk in MACEs after first prescription of SABA was observed in a nested case–control analysis in the UK, compared with short-acting muscarinic antagonist (SAMA) [51]. Frequent SABA use may also reflect and contribute to a vicious cycle in COPD, whereby increased reliance on rescue bronchodilation is associated with a higher risk of exacerbations [52], and these exacerbations, in turn, are well-established triggers of acute cardiovascular events, amplifying overall cardiovascular risk. Short-acting muscarinic antagonists, such as ipratropium, are commonly administered, often via nebulization, in combination with short-acting beta-2-agonists to enhance bronchodilation [53]. Evidence from clinical studies suggests that combining bronchodilators of different classes improves lung function and symptom control, although their independent cardiovascular effects remain less well characterized, with available data showing no consistent increase in cardiovascular events [54].

### 5.2. Systemic Corticosteroids

Systemic corticosteroids reduce treatment failure and shorten hospital stay, while improving lung function and dyspnea during COPD exacerbations [55,56], but their metabolic and hemodynamic effects may adversely influence cardiovascular risk in an already vulnerable population [57]. Glucocorticoids cause fluid retention and sodium imbalance due to their mineralocorticoid activity, which may promote hypertension and precipitate heart failure decompensation [58], as well as corticosteroid-induced hyperglycemia, contributing to acute metabolic stress and endothelial dysfunction [59]. However, these side effects are more commonly associated with prolonged or higher-dose exposure rather than short-course therapy typically used in this setting [60]. Increased renal potassium excretion may lead to hypokalemia, facilitating arrhythmogenesis [58], particularly in the presence of concomitant beta-2 agonist therapy [61,62]. Glucocorticoid exposure has also been associated with an increased risk of both venous and arterial thrombotic events, likely mediated by procoagulant shifts in hemostatic factors, enhanced platelet activity, and endothelial dysfunction [63]. However, translating these pathophysiological mechanisms into clinical cardiovascular risk is not straightforward, as recent evidence indicates a heterogenous association between glucocorticoid exposure and acute cardiovascular events, shaped by dose, duration, and individual patient vulnerability [64]. In a nationwide registry-based cohort study, Baunsgaard-Pedersen et al. reported no significant association between short-term oral corticosteroid (OCS) use and the risk of major adverse cardiovascular events within one year in outpatients with COPD, compared with antibiotic therapy alone. Repeated short-term OCS exposure was likewise not associated with an increased risk in MACE, as no cumulative dose–response relationship was observed [65]. In contrast, a large meta-analysis of 43 studies reported that glucocorticoid exposure was associated with increased cardiovascular risk overall, including MACEs, coronary heart disease, and heart failure, and further suggested a dose–response relationship, with MACE risk rising by 10% for each additional gram of cumulative exposure [57]. However, the included populations were heterogeneous and not specific to COPD, limiting the direct applicability of these findings to exacerbation settings. These considerations have led to increasing interest in individualized corticosteroid strategies aimed at balancing therapeutic benefit against systemic risk. In the outpatient setting, a multicenter, randomized controlled trial in primary care evaluated a biomarker-guided approach in which oral prednisolone was prescribed according to point-of-care blood eosinophil counts. Patients with higher eosinophil levels received prednisolone, whereas those with low counts were treated with placebo. This strategy achieved similar clinical outcomes to standard care while reducing systemic corticosteroid exposure [66]. Ongoing randomized controlled trials are expected to further define the role of eosinophil-guided corticosteroid therapy in optimizing treatment and reducing potential systemic and cardiovascular risk [67].

### 5.3. Antibiotic Therapy

According to current guidelines, antibiotics are recommended during COPD exacerbations in patients presenting with increased sputum purulence or fever, particularly when accompanied by increased sputum volume and dyspnea, or in those requiring mechanical ventilation [3]. Biomarkers such as C-reactive protein and procalcitonin have been investigated as tools to distinguish bacterial from non-bacterial exacerbations, with moderate evidence supporting their diagnostic utility [68]. Antibiotic therapy should provide coverage against the most common pathogens [69] and be tailored to local antimicrobial resistance patterns, with commonly used options including aminopenicillins combined with clavulanic acid, macrolides, or tetracyclines [70]. Emerging evidence suggests that shorter treatment durations, typically around five days, may be as effective as longer courses, despite the lack of clear consensus on optimal duration [71,72]. The benefits of certain antibiotics, particularly macrolides, extend beyond antimicrobial activity, potentially involving modulation of airway inflammation and immune responses [73]. At the same time, repeated or prolonged antibiotic exposure has been associated with antimicrobial resistance and alterations in airway microbial composition [74]. Beyond their role in the management of exacerbations, certain antibiotic classes have shown efficacy in preventing exacerbations in patients with COPD, with macrolides showing greater benefit than tetracyclines or fluoroquinolones [75]. Azithromycin and erythromycin have been shown to reduce exacerbation frequency, prolong the time to first exacerbation, and decrease bacterial load [76]. However, the use of this class raises important safety concerns, particularly regarding cardiovascular risk. Macrolides have been associated with QT interval prolongation, which may predispose susceptible individuals to ventricular arrhythmias, including torsades de pointes [77]. Kono et al. conducted a pharmacovigilance analysis evaluating the association between individual macrolides and cardiovascular adverse events. Azithromycin showed the strongest association with ventricular tachyarrhythmias (ROR 4.2; CI 95% 3.88–4.62), followed by clarithromycin (ROR 3.6; CI 95% 3.17–4.05) and erythromycin (ROR 3.5; CI 95% 3.07–4.09). Notably, azithromycin demonstrated additional associations with non-arrhythmic cardiovascular events, including hypertension, heart failure, and bleeding-related abnormalities [78]. Similar findings have been described in a large-scale meta-analysis, supporting an association between macrolide use and increased risk of ventricular arrhythmias, sudden cardiac death, and cardiovascular mortality, in the absence of a clear effect on overall mortality [79]. However, these findings are not consistent across the literature, as a large systematic review did not demonstrate an increased risk of arrhythmia or cardiovascular mortality with this drug class, and identified only a modest association with myocardial infarction [80]. Furthermore, observational data suggest that the apparent cardiovascular risk may be substantially attenuated after adjustment for patient characteristics and comorbidities, becoming non-significant in fully adjusted models [81]. This raises the possibility that the reported cardiovascular risk may reflect underlying patient vulnerability or indication-related bias, rather than a direct pharmacological effect.

### 5.4. Oxygen and Ventilatory Support

Beyond pharmacological management, supportive interventions used during COPD exacerbations may also have important cardiovascular implications. Among these, oxygen therapy is widely used in the acute setting, yet both hypoxemia and its correction can influence cardiovascular physiology and outcomes [82,83]. Hypoxemia represents a potent trigger of cardiovascular stress, promoting sympathetic activation, tachycardia, and systemic vasoconstriction, while hypoxic pulmonary vasoconstriction increases right ventricular afterload and may precipitate or worsen right heart dysfunction [84,85]. In addition, reduced arterial oxygen content may impair myocardial oxygen supply, contributing to supply–demand mismatch and increasing the risk of myocardial ischemia, particularly in patients with underlying coronary artery disease [32,41]. However, the correction of hypoxemia through supplemental oxygen is not without potential adverse effects. Excessive oxygen administration may lead to hypercapnia in susceptible patients, through mechanisms including ventilation–perfusion mismatch and reduced hypoxic respiratory drive [86]. Moreover, hyperoxia may exert adverse cardiovascular effects through increased production of reactive oxygen species, promoting oxidative stress and endothelial dysfunction, and contributing to vasoconstriction, reduced coronary blood flow, and impaired microcirculatory perfusion [87]. Alongside oxygen therapy, ventilatory support strategies, including non-invasive ventilation (NIV) and high-flow oxygen (HFO), are commonly employed during COPD exacerbations and can further modify cardiovascular dynamics. In stable COPD, compensatory mechanisms such as dynamic hyperinflation and accessory muscle recruitment help maintain ventilation. However, these fail during exacerbations, leading to tachypnea, reduced tidal volumes, hypercapnia, and respiratory acidosis with increased work of breathing. Bilevel NIV delivers distinct inspiratory and expiratory pressures, with the pressure gradient determining tidal volume and, consequently, alveolar ventilation [88]. By augmenting tidal volume and supporting ventilation, NIV improves carbon dioxide clearance and corrects respiratory acidosis, while simultaneously reducing the work of breathing and respiratory muscle fatigue, thereby preventing further clinical deterioration [89]. The physiological effects of non-invasive ventilation extend beyond gas exchange, as it reduces left ventricular transmural pressure, thereby lowering afterload and facilitating ventricular ejection, with potential improvement in stroke volume, particularly in patients with coexisting left ventricular dysfunction [90]. In addition, NIV has been associated with reductions in heart rate and cardiac workload, likely reflecting improved oxygenation and reduced respiratory distress [91]. Overall, evidence suggests that NIV improves outcomes in exacerbations of COPD, with reductions in mortality, intubation rates, and hospital length of stay, supporting its widespread use in clinical practice [89,92]. High-flow nasal oxygen (HFNO) may be considered in selected patients, particularly when NIV is poorly tolerated, with evidence indicating comparable efficacy in mild-to-moderate hypercapnic exacerbations and improved patient comfort [93].

### 5.5. Maintenance Therapy

Following the acute phase, long-acting bronchodilators, including beta-2-agonists (LABAs) and muscarinic antagonists (LAMAs), are central to maintenance therapy and have been shown to reduce the risk of recurrent exacerbation [94]. Their impact on cardiovascular outcomes remains an area of ongoing investigation. Observational studies have suggested a possible increase in cardiovascular risk shortly after treatment initiation, particularly within the first month [95]. However, this signal has not been consistently confirmed, with several analyses reporting neutral effects during longer-term use, underscoring the heterogeneity of the available evidence [96,97]. In contrast, LAMAs are generally regarded as having a favorable cardiovascular profile, with meta-analytic data from randomized trials showing no increased risk of cardiovascular events compared with placebo. In addition, LAMAs improve lung function and reduce exacerbation frequency, which may indirectly mitigate cardiovascular risk [98]. Evidence from randomized clinical trials indicates that dual LABA/LAMA therapy provides improvements in airflow limitation, symptom burden, and disease control compared with monotherapy, while also reducing reliance on rescue medication and lowering the risk of clinical deterioration, without an increase in serious adverse events [99]. In a real-world study by Suissa et al., adding a second long-acting bronchodilator (either LABA or LAMA) to existing monotherapy did not increase the risk of major cardiovascular events, including myocardial infarction, stroke, or arrhythmia. However, a modestly elevated risk of heart failure was observed, highlighting the need for careful monitoring in high-risk patients. These findings support the overall cardiovascular safety of combination long-acting bronchodilator therapy while emphasizing caution regarding heart failure [100].

Escalation to triple therapy has been shown to significantly reduce the rate of moderate and severe exacerbations compared with dual therapy, while also improving symptom burden and health-related quality of life [3]. These benefits appear consistent across patient subgroups, although they may be more pronounced in former smokers, potentially reflecting greater responsiveness to inhaled corticosteroids (ICS) [101]. Evidence from ETHOS trial demonstrates that triple therapy with budesonide/glycopyrrolate/formoterol is associated with a significant reduction in all-cause mortality compared with dual bronchodilator therapy. This effect appears to be dependent on the dose of the inhaled corticosteroid component, with a significant mortality reduction observed only at higher doses, and cannot be explained solely by a reduction in exacerbations, suggesting additional mechanisms of benefit. Cardiovascular causes accounted for a substantial proportion of deaths, with fewer events observed in patients receiving inhaled corticosteroid-containing regimens. These findings support the hypothesis that triple therapy may exert beneficial systemic effects, potentially through modulation of inflammation and improvement of cardiopulmonary interactions [102]. Notably, a large metanalysis conducted by Hammadi et al. demonstrated no significant differences between single triple-inhaler therapy and LABA/ICS or LAMA/LABA regimens regarding major cardiovascular event, while supporting previous observations on mortality [103]. Taken together, these findings indicate that triple therapy may provide both respiratory and systemic benefits, including potential reductions in mortality and cardiovascular risk. Nonetheless, the absence of clear superiority over ICS-containing dual regimens and the heterogeneity of available evidence emphasize the need for careful, individualized treatment selection [104].

Beyond inhaled therapies, roflumilast may be considered in patients with severe COPD associated with chronic bronchitis and recurrent exacerbations despite optimized maintenance treatment. As an oral phosphodiesterase-4 (PDE-4) inhibitor, it reduces airway and systemic inflammation and has been shown to decrease exacerbation frequency [105]. Given the established association between exacerbations, systemic inflammation, and cardiovascular events in COPD, these effects may have favorable cardiovascular implications [44]. However, evidence regarding the direct effects of roflumilast on cardiovascular outcomes remains limited, and further studies are required to clarify its role in the prevention of cardiovascular complications in COPD.

Recent advances in the understanding of COPD endotypes have led to the development of biologic therapies targeting type 2 inflammation. Dupilumab, a monoclonal antibody directed against the interleukin-4 receptor alpha (IL-4Rα), inhibits the interleukin-4/interleukin-13 (IL-4/IL-13) signaling pathway and has demonstrated promising results in patients with eosinophilic COPD. In the phase III BOREAS trial, patients with blood eosinophil counts ≥ 300 cells/µL who remained at high risk of exacerbations despite background inhaled triple therapy experienced a lower annualized rate of moderate or severe exacerbations compared with placebo (0.78 versus 1.10), along with significant improvements in lung function and quality of life [106]. These findings support a more personalized therapeutic approach in selected COPD populations and highlight the importance of inflammatory endotyping in disease management. While the cardiovascular implications of biologic therapy in COPD remain largely unexplored, modulation of the IL-4/IL-13 axis may influence pathways involved in vascular inflammation and endothelial dysfunction, although its impact on cardiovascular outcomes has yet to be established [107].

**Table 2:** Therapeutic strategies in COPD exacerbations and cardiovascular considerations.

Therapeutic Category	Intervention/Concept	Clinical Effects/Benefits	Cardiovascular Considerations/Risks
Short-acting bronchodilators	SABAs	First-line therapy with rapid bronchodilation in moderate–severe exacerbations [3]	High doses may cause systemic absorption with tachycardia; frequent use associated with increased acute cardiovascular events [48,50]
Combination bronchodilation	SABA + SAMA	Improved lung function and symptom control [53,54]	No consistent increase in cardiovascular events, though data remain limited [54]
Systemic corticosteroids	Use in exacerbations	Reduce treatment failure, shorten hospital stay, and improve lung function and dyspnea [55,56]	May increase cardiovascular risk via fluid retention and hypertension, hyperglycemia and endothelial dysfunction, and prothrombotic effects [57,58,59,64]
Antibiotic therapy	Indications and regimens	Recommended in selected patients; common agents include aminopenicillins/clavulanate, macrolides, tetracyclines; short courses (~5 days) are generally effective [3,70,71,72]	Certain agents, particularly macrolides, have been associated with QT interval prolongation and increased risk of ventricular arrhythmias; observed cardiovascular risk may be influenced by patient comorbidities and indication bias [77,81]
Oxygen therapy	Controlled oxygen administration	Essential for correction of hypoxemia [82,83]	Hypoxemia induces sympathetic stress and right heart strain, while excessive oxygen may cause hypercapnia and hyperoxia-related oxidative stress, vasoconstriction, and reduced coronary perfusion [83,84,85,86,87]
Maintenance therapy	LABAs and LAMAs	Reduce risk of recurrent exacerbations [94]	Dual therapy does not increase major CV events but may slightly raise heart failure risk [100]
	LABA/LAMA/ICS	Reduces exacerbation frequency, improves symptom control and quality of life [3]	Fewer cardiovascular events when compared to LABA/LAMA regimens, with effects depending on ICS dosage [102]; Reduction in all-cause mortality [102,103]
	Roflumilast (PDE-4 inhibitor)	Reduces exacerbation frequency [105]	Potential indirect cardiovascular benefit through inflammation and exacerbation reduction; its effect on cardiovascular event prevention has not been clearly defined [44]
	Dupilumab (anti-IL-4Rα monoclonal antibody)	Reduces exacerbations and improves lung function in eosinophilic COPD [106]	Modulation of the IL-4/IL-13 axis may influence vascular inflammation and endothelial dysfunction; effects on cardiovascular outcomes have yet to be established [107]

## 6. Cardiovascular Therapies: Missed Opportunities

While COPD-directed therapies may indirectly influence cardiovascular outcomes by reducing exacerbations and improving systemic physiology, they do not specifically target the underlying cardiovascular risk. Given the high burden of cardiovascular comorbidities in this population, this highlights a critical gap in care, where established cardioprotective therapies remain suboptimal in a high-risk population [108].

Beta-blockers are insufficiently prescribed in patients with COPD despite clear cardiovascular indications, largely due to concerns regarding potentially respiratory adverse effects. However, evidence indicates that long-term use of cardioselective beta-1-blockers does not result in clinically meaningful declines in lung function and does not impair the bronchodilator response to inhaled beta-2-agonists, even in patients with more advanced disease. Differences between agents exist, with cardioselective drugs, particularly bisoprolol, generally better tolerated than non-cardioselective beta-blockers [109]. Real-world data show that patients with coexisting heart failure and COPD are significantly less likely to receive beta-blockers compared with those with heart failure alone, even when guideline-directed therapy is indicated [110]. In a large nationwide cohort of patients hospitalized for COPD exacerbations with coexisting heart failure, only 11.9% received guideline-directed therapy for both conditions. Guideline-directed heart failure therapy was associated with a 38% reduction in in-hospital mortality, while COPD therapy conferred a similar benefit (40% reduction), with the lowest mortality observed in patients receiving both [111]. Evidence from a recent prospective cohort study further supports the safety of beta-blockers in high-risk clinical settings. Among patients with COPD hospitalized for acute myocardial infarction, beta-blockers were prescribed at discharge in 86.7% of cases. Beta-blocker therapy was not associated with an increased risk of all-cause mortality, cardiovascular events, or respiratory complications, with no difference observed in the composite outcome of death, hospitalization, or revascularization [112]. Taken together, these findings suggest that concerns regarding respiratory safety should no longer justify withholding beta-blockers in patients with COPD and standard cardiovascular indications.

This therapeutic gap extends beyond beta-blockers. Even when cardiovascular disease is present, antiplatelet agents, statins, and renin-angiotensin system inhibitors are not consistently prescribed, despite well-established benefits in secondary prevention and heart failure management, indicating that exacerbations are not routinely used as an opportunity to initiate or optimize cardioprotective therapy [113].

Thromboembolic complications represent another important cardiovascular consideration in COPD. Systemic inflammation, hypoxemia, endothelial dysfunction, and coagulation abnormalities contribute to a prothrombotic state that may increase susceptibility to venous thromboembolism and pulmonary embolism in this population [114]. Although these observations support a biological rationale for anticoagulation, current evidence does not support routine anticoagulation solely on the basis of COPD. In the SLICE randomized clinical trial, a systematic strategy of pulmonary embolism screening during hospitalization for COPD exacerbation did not significantly reduce the composite outcome of venous thromboembolism, COPD readmission, or death compared with usual care [115]. Therefore, current evidence supports a targeted approach focused on the prompt recognition of thromboembolic complications and the use of anticoagulant therapy when established indications are present, rather than routine anticoagulation in all patients with COPD exacerbations.

Statins illustrate the uncertainty surrounding cardiovascular therapies in COPD. Although observational studies and some randomized data suggest a reduction in exacerbations [116], results are inconsistent, and larger trials have not confirmed a benefit [117]. Notably, these effects occur without meaningful changes in lung function or systemic inflammation. Overall, current evidence does not support a COPD-specific role for statins beyond their established cardiovascular indications [118].

Among emerging therapeutic strategies, glucagon-like peptide-1 receptor agonists (GLP-1RAs) have attracted increasing interest because of their anti-inflammatory, metabolic, and weight-reducing effects. In a large population-based cohort of patients with COPD and type 2 diabetes, GLP-1RA use was associated with a 30% lower risk of severe exacerbation compared with sulfonylureas [119]. Supporting these observations, a randomized placebo-controlled trial of liraglutide in patients with obesity and COPD demonstrated improvements in forced vital capacity, diffusion capacity, and COPD-related health status, while effects on airflow limitation (FEV1 and FEV1/FVC) and systemic inflammatory markers were not significant [120]. A recent meta-analysis including 62,678 patients with obstructive lung disease and type 2 diabetes further demonstrated that GLP-1RA therapy was associated with significantly lower exacerbation rates compared with both sulfonylureas and dipeptidyl peptidase-4 inhibitors. While the pooled analysis included both asthma and COPD populations, the findings were consistent with those observed in COPD-specific studies [121]. Given their established cardiometabolic benefits and emerging respiratory effects, GLP-1RAs may offer a unique opportunity to address both pulmonary and cardiovascular risk in selected patients with COPD.

## 7. Clinical Implications and Future Directions

The close relationship between COPD exacerbations and cardiovascular events has direct consequences for clinical practice. Exacerbations should be regarded not only as episodes of respiratory deterioration but also as periods during which underlying cardiovascular disease may become clinically relevant or newly detectable. A more structured cardiovascular assessment during exacerbations improves the detection of previously unrecognised cardiac disease. In a recent pilot randomised study, a systematic evaluation strategy, including echocardiographic evaluation, coronary artery calcium scoring, ambulatory rhythm monitoring, and laboratory assessment of cardiometabolic risk, identified a markedly higher proportion of new cardiovascular diagnoses (74%) compared with usual care (19%) and resulted in more frequent initiation of appropriate treatment [122]. These findings challenge the current symptom-driven approach to cardiovascular investigation and support the concept of proactive screening in high-risk patients, particularly during hospitalization, which represents a critical window for intervention. In parallel, limitations of conventional cardiovascular risk stratification in COPD highlight the need for improved tools. Current clinical prediction models remain insufficient in this setting. A recent systematic review and meta-analysis found that no validated models exist for predicting combined cardiopulmonary risk in patients with COPD, while available models for cardiovascular events, exacerbations, or mortality are limited by small sample sizes, lack of external validation, and high risk of bias [123]. Combining traditional risk scores with imaging-based markers, such as coronary artery calcium, may enhance risk prediction and facilitate identification of high-risk individuals. Opportunistic evaluation of coronary artery calcium on routine chest CT imaging offers a practical and readily available option for cardiovascular risk stratification in this population [124].

In addition to imaging, circulating biomarkers may further refine risk assessment. The neutrophil-to-lymphocyte ratio, derived from routine blood tests, reflects systemic inflammatory activity and has been associated with an increased risk of cardiovascular events following exacerbations, with prognostic relevance extending up to one year [125]. Biomarkers reflecting myocardial injury provide complementary information. Cardiac troponin elevation was observed during exacerbations, even in the absence of confirmed acute coronary syndromes, and has been linked to increased mortality and adverse clinical outcomes, reflecting a combination of myocardial stress, hypoxia, and underlying cardiovascular disease rather than isolated ischemic events [126]. Growth differentiation factor-15 (GDF-15), a stress-responsive cytokine induced by inflammatory, oxidative, and hypoxic pathways, has also been associated with an increased risk of MACE and all-cause mortality in patients hospitalized for ECOPD. Its association appears to reflect systemic and cardiovascular processes rather than COPD-specific outcomes, suggesting that it captures a broader burden of physiological stress during exacerbation [127].

Improving cardiovascular outcomes in patients with COPD will likely depend not on a single tool, but on integrating clinical assessment, imaging, and biomarker profiling into a coherent strategy capable of identifying high-risk individuals and guiding appropriate management during critical periods following exacerbation.

### The Mission of Pulmonologists and Cardiologists During the Vulnerable Window

From a practical standpoint, pulmonologists are often the first to interpret complex clinical presentations in which respiratory and cardiac features overlap. As such, their contribution lies less in establishing definitive cardiovascular diagnoses and more in identifying patterns that warrant further evaluation. Subtle deviations from the expected course of an exacerbation, disproportionate symptom burden, or atypical responses to standard therapy may all suggest a concurrent cardiovascular component and should prompt additional investigation or specialist input [128]. Given the clinical impact of exacerbations on subsequent outcomes, risk stratification becomes an important component of this role. In practice, this involves contextualizing the current episode within the patient’s broader clinical trajectory, where factors such as prior exacerbation frequency, underlying disease severity, and comorbidity burden provide important signals regarding future vulnerability [129]. In this way, the pulmonologist’s role extends from pattern recognition to early risk stratification, effectively shaping clinical decisions, including the need for closer monitoring, treatment intensification, or timely cardiology involvement.

In contrast, cardiology involvement becomes relevant in the stabilization and post-acute phase, where the focus shifts from immediate symptom control to risk modification and prevention of recurrent events. Cardiology assessment is often guided by specific clinical triggers rather than systematic screening. Symptoms such as chest pain suggestive of angina, palpitations of unclear origin, or dyspnea that exceeds what would be expected based on lung function should prompt referral and further evaluation [109]. Once cardiovascular disease is identified, the implementation of guideline-directed therapy remains essential, despite concerns regarding tolerability. Beta-blockers, although frequently underused due to concerns about bronchoconstriction, should not be withheld when indicated. Additional therapies used in heart failure, including sodium-glucose cotransporter-2 inhibitors and diuretics, contribute to cardiovascular stabilization, particularly through the reduction in congestion, which may alleviate overlapping respiratory symptoms [130]. Therefore, the role of the cardiologist lies not only in initiating appropriate therapy but also in balancing cardiovascular benefit against potential pulmonary impact.

Rather than duplicating evaluations across specialties, a more effective model may therefore rely on aligning their contributions within a coordinated care pathway. Pulmonology-led acute management would incorporate targeted cardiovascular awareness, while cardiology-led follow-up would focus on consolidation and optimization of cardiovascular care.

## 8. Limitations

This review has several limitations. First, as a narrative review, study selection was guided by relevance to the predefined themes rather than by a formal systematic review protocol, which may introduce selection bias. Second, the available literature is characterized by substantial heterogeneity in study design, patient populations, definitions of COPD exacerbations, and cardiovascular outcomes, limiting direct comparisons across studies. Third, much of the evidence linking COPD exacerbations to cardiovascular events originates from observational studies and registry-based analyses, which are susceptible to residual confounding and cannot establish causality. Finally, evidence regarding several emerging therapeutic strategies remains limited, with relatively few studies specifically evaluating their impact on cardiovascular outcomes following COPD exacerbations.

## 9. Conclusions

COPD exacerbations mark a period of disproportionate cardiovascular risk that is not fully captured by current clinical approaches. While the temporal association between exacerbations and major cardiovascular events is well established, its implications for routine care remain insufficiently translated into practice. A central challenge lies between recognition and action. Cardiovascular events are frequently overlooked in the context of acute respiratory deterioration, and opportunities to initiate or optimize cardioprotective therapies are often missed. At the same time, treatments used during exacerbations may influence cardiovascular risk in complex and sometimes opposing ways, further underscoring the need for a more integrated approach. Rather than being viewed as isolated respiratory episodes, exacerbations should be considered clinically meaningful events that signal a need for broader risk reassessment. In this context, the early post-exacerbation period offers a practical opportunity to identify previously unrecognized cardiovascular disease and to implement targeted interventions. Future research should aim to better define risk stratification strategies specific to this setting and to clarify how clinical, imaging, and biomarker-based tools can be incorporated into routine care. Bridging the gap between evidence and implementation may be essential to reducing the persistent burden of cardiovascular morbidity and mortality in patients with COPD, and will likely depend on closer integration between pulmonology and cardiology care.

## Data Availability

No new data were created or analyzed in this study. Data sharing is not applicable to this article.

## Abbreviations

The following abbreviations are used in this manuscript:

COPD	Chronic Obstructive Pulmonary Disease
MACE	Major Adverse Cardiovascular Event
GOLD	Global Initiative for Chronic Obstructive Lung Disease
ECOPD	Exacerbations of Chronic Obstructive Pulmonary Disease
AMI	Acute Myocardial Infarction
AF	Atrial Fibrillation
HF	Heart Failure
PE	Pulmonary Embolism
CRP	C-reactive Protein
IL-6	Interleukin-6
IL-8	Interleukin-8
ROS	Reactive Oxygen Species
NO	Nitric Oxide
HIF-1	Hypoxia-Inducible Factor-1
PMCs	Platelet–Monocyte Complexes
SABAs	Short-Acting Beta-2 Agonists
SAMAs	Short-Acting Muscarinic Antagonists
OCS	Oral Corticosteroid
NIV	Non-Invasive Ventilation
HFO	High Flow Oxygen
LABAs	Long-Acting Beta-2 Agonists
LAMAs	Long-Acting Muscarinic Antagonists
ICS	Inhaled Corticosteroid
PDE-4	Phosphodiesterase-4
IL-4	Interleukin-4
IL-13	Interleukin-13
GLP-1RA	Glucagon Like Peptide-1 Receptor Agonist
FEV1	Forced Expiratory Volume in 1 Second
FVC	Forced Vital Capacity
